# Tetanus in Serbia, 2015–2025: Epidemiological Overview and Lessons from Two Pediatric Cases

**DOI:** 10.3390/diseases14100385

**Published:** 2026-10-20

**Authors:** Gordana Kovacevic, Vesna Stijovic, Branko Kovacevic, Miro Parezanovic, Sofija Popovic, Marija Simic, Stefan Kotlajic, Gordana Petrovic, Srdjan Pasic

**Affiliations:** 1 Department of Neurology, Mother and Child Health Care Institute of Serbia “Dr Vukan Čupić”, 11070 Belgrade, Serbia; 2 Faculty of Medicine, University of Belgrade, 11070 Belgrade, Serbia; srdjan.pasic@med.bg.ac.rs; 3 Institute of Public Health of Belgrade, 11000 Belgrade, Serbia; vesna.stijovic@zdravlje.org.rs; 4 Pediatric Intensive Care Unit, Mother and Child Health Care Institute of Serbia “Dr Vukan Čupić”, 11070 Belgrade, Serbia; branko.kovacevic@imd.org.rs (B.K.); mlparezanovic@yahoo.com (M.P.); 5 Pediatric Clinic, Mother and Child Health Care Institute of Serbia “Dr Vukan Čupić”, 11070 Belgrade, Serbia; sofija.popovic@imd.org.rs (S.P.); marija.simic@imd.org.rs (M.S.); 6 Department of Immunology, Mother and Child Health Care Institute of Serbia “Dr Vukan Čupić”, 11070 Belgrade, Serbia; stefan.kotlajic@gmail.com (S.K.); gordana.petrovic.im@gmail.com (G.P.)

**Keywords:** pediatric tetanus, trismus, autonomic instability, human tetanus immunoglobulin tetanus toxoid-containing vaccines, vaccination coverage, vaccination hesitancy

## Abstract

Background: Tetanus is a vaccine-preventable disease that has become rare in countries with sustained immunization programs. However, unvaccinated children remain at risk of severe disease. We provide an epidemiological overview of tetanus in Serbia and describe two cases of generalized tetanus in children. Methods: We retrospectively reviewed tetanus surveillance data and immunization coverage reports in Serbia for 2015–2025. Tetanus cases were recorded under ICD-10 code A35. We analyzed demographics, incidence, outcomes, case-fatality, and tetanus-containing vaccine coverage. Two pediatric cases were reviewed for clinical presentation, diagnostic challenges, management, and outcomes. Results: Fourteen tetanus cases were reported during 2015–2025 (annual incidence, 0–0.06 per 100,000 population), including two children. Most cases occurred among women aged >60 years; the overall case-fatality proportion was 42.9%. Primary vaccination coverage ranged from 90.8% to 95.1%, while recommended revaccination coverage remained below the 95% target. Both pediatric patients had received only BCG and hepatitis B vaccination at birth with no subsequent routine immunization due to parental neglect or refusal. The 18-month-old boy presented with fever, otorrhea, and recurrent episodes of generalized stiffening and was initially evaluated for meningoencephalitis. The emergence of trismus, opisthotonus, and neck muscle rigidity led to the diagnosis of tetanus. The 6-year-old boy presented with odynophagia and involuntary tongue biting, followed by progressive trismus and limb spasms. Tetanus was suspected based on the clinical findings and absent immunization. He developed respiratory failure and autonomic instability. Both patients recovered following tetanus immunoglobulin, antimicrobial therapy, and supportive care. Conclusion: Although tetanus remains rare in Serbia and predominantly affects older adults, unvaccinated children remain at risk of severe disease. Atypical presentations may delay diagnosis, emphasizing the importance of early recognition and timely treatment. Maintaining high vaccination coverage and ensuring catch-up immunization remain essential for prevention.

## 1. Introduction

Tetanus is a potentially life-threatening but entirely vaccine-preventable disease characterized by generalized muscle rigidity and painful spasms [1,2]. Owing to widespread immunization programs, pediatric tetanus has become exceedingly rare in many countries, including Serbia [1,2,3,4,5,6,7,8,9]. Nevertheless, sporadic cases continue to occur, particularly among children who have not received recommended tetanus-containing vaccines [10,11]. Missed vaccination opportunities, due to social vulnerability, limited access to healthcare, or parental vaccine refusal, may contribute to disease occurrence [12,13]. In addition to inadequate immunization, tetanus may occur following traumatic wounds, particularly deep or puncture wounds and wounds contaminated with soil or foreign material, especially when appropriate wound care and post-exposure prophylaxis are not provided [14,15].

Tetanus results from the action of tetanospasmin, a potent neurotoxin produced by *Clostridium tetani*, leading to muscle rigidity, painful spasms, and autonomic dysfunction [2,3].

Although generalized tetanus has a characteristic clinical phenotype in its advanced stages, early manifestations are frequently nonspecific and may mimic more common neurological, otorhinolaryngological, or dental conditions, particularly in children [2,3,4,14,15]. As no laboratory test can reliably confirm or exclude tetanus, the diagnosis remains primarily clinical and should be considered in patients with compatible neuromuscular symptoms, a history of a wound or potential portal of entry, and lack of tetanus immunization [1,2,10,16]. Failure to recognize evolving symptoms or to appreciate the significance of vaccination status may delay initiation of appropriate treatment. Severe generalized tetanus may be complicated by respiratory failure and autonomic dysfunction and may require prolonged intensive care. Management requires prompt administration of human tetanus immunoglobulin, antimicrobial therapy, control of muscle spasms, and supportive care [2,4,16,17]. Despite advances in treatment and intensive care, including ventilatory support, severe tetanus may still result in substantial mortality, largely because of severe autonomic dysfunction, cardiovascular instability, and complications associated with prolonged critical illness [2,4].

Although pediatric tetanus is rare in countries with established immunization programs, each case represents a potentially preventable event with important clinical and public health implications. Combining clinical case descriptions with epidemiological data can highlight both diagnostic challenges and gaps in immunization.

The aim of this study was to describe the only two pediatric cases of generalized tetanus reported in Serbia during the 2015–2025 period, both occurring in unvaccinated children, and to characterize the circumstances underlying their lack of vaccination. The cases were further placed in the context of national tetanus epidemiological trends and childhood tetanus immunization coverage, with particular emphasis on age distribution and clinical outcomes.

## 2. Materials and Methods

### 2.1. Study Design

This retrospective descriptive study comprised two complementary components: (1) epidemiological overview of tetanus surveillance data and childhood tetanus-containing vaccine coverage in Serbia from 2015 to 2025, and (2) a retrospective descriptive case series of two pediatric patients with generalized tetanus treated at a tertiary referral center between 2023 and 2025. These two patients represented the only pediatric tetanus cases reported in Serbia during the study period.

#### 2.1.1. National Tetanus Surveillance and Vaccination Coverage Data

National tetanus surveillance data for 2015–2025 were retrospectively reviewed using published annual surveillance reports [4,5,6,7,8,9] and official surveillance data provided by the Institute of Public Health of Serbia “Dr Milan Jovanović Batut”. Tetanus cases were recorded in the surveillance database under the International Classification of Diseases, 10th Revision (ICD-10), code A35 (Tetanus alius) [18]. The epidemiological overview included demographic characteristics (age and sex), geographic distribution (Central Serbia and Vojvodina), annual incidence, case outcome, and overall case-fatality proportion. Data obtained from the annual reports are presented descriptively as absolute numbers, percentages, and incidence rates per 100,000 population.

Data on childhood vaccination coverage with tetanus-toxoid-containing vaccines for the period 2015–2025 were obtained from published official tables and reports, supplemented by official data provided by the Institute of Public Health of Serbia “Dr Milan Jovanović Batut”. Primary immunization refers to completion of a three-dose primary series of a tetanus-toxoid-containing vaccine during the first year of life. The first, second, and third revaccinations consist of one booster dose each, scheduled during the second year of life, before entry into the first grade of primary school, and in the final grade of primary school, respectively. According to the methodology of the source reports, coverage is expressed as the number of children who completed the corresponding primary series or received the scheduled booster dose divided by the number of children planned to receive that immunization in the reporting year, multiplied by 100. The denominator is therefore specific to each immunization category and reporting year [19,20,21].

#### 2.1.2. Clinical Data from Two Pediatric Tetanus Cases

Clinical, laboratory, diagnostic, treatment, and outcome data for the two pediatric patients were retrospectively extracted from medical records. Both patients were diagnosed with generalized tetanus based on clinical presentation and appropriate evaluation of alternative diagnoses. The two pediatric cases were presented narratively.

### 2.2. Ethical Approval

Ethical approval was obtained from the Institutional Ethics Committees of Mother and Child Health Care Institute of Serbia “Dr Vukan Čupić” (protocol code 8/133, date of approval 2 November 2025), in accordance with the Declaration of Helsinki. Written informed consent was obtained from the parents or legal guardians of all patients.

## 3. Results

### 3.1. Epidemiological Characteristics of Reported Tetanus Cases in Serbia, 2015–2025

A total of 14 tetanus cases were reported in Serbia during the 2015–2025 study period (Table 1). No tetanus cases were reported in 2018–2020 or 2024.

Overall, females accounted for 71.4% of all reported cases, while males accounted for 28.6%. Most cases (11/14) occurred among individuals aged ≥60 years, whereas three cases were reported in younger age groups, including one patient aged 20–29 years and two pediatric cases aged 1–9 years. Most cases were reported from Central Serbia (12/14, 85.7%), while two cases (14.3%) were reported from Vojvodina. Vaccination status was unknown for 12 patients, whereas both pediatric cases were unvaccinated against tetanus. Six patients died during the study period, corresponding to an overall case fatality proportion of 42.9% (Table 1). All fatal outcomes occurred among patients aged ≥60 years, while no tetanus-associated deaths were reported from 2021 onward.

**Figure 1:**
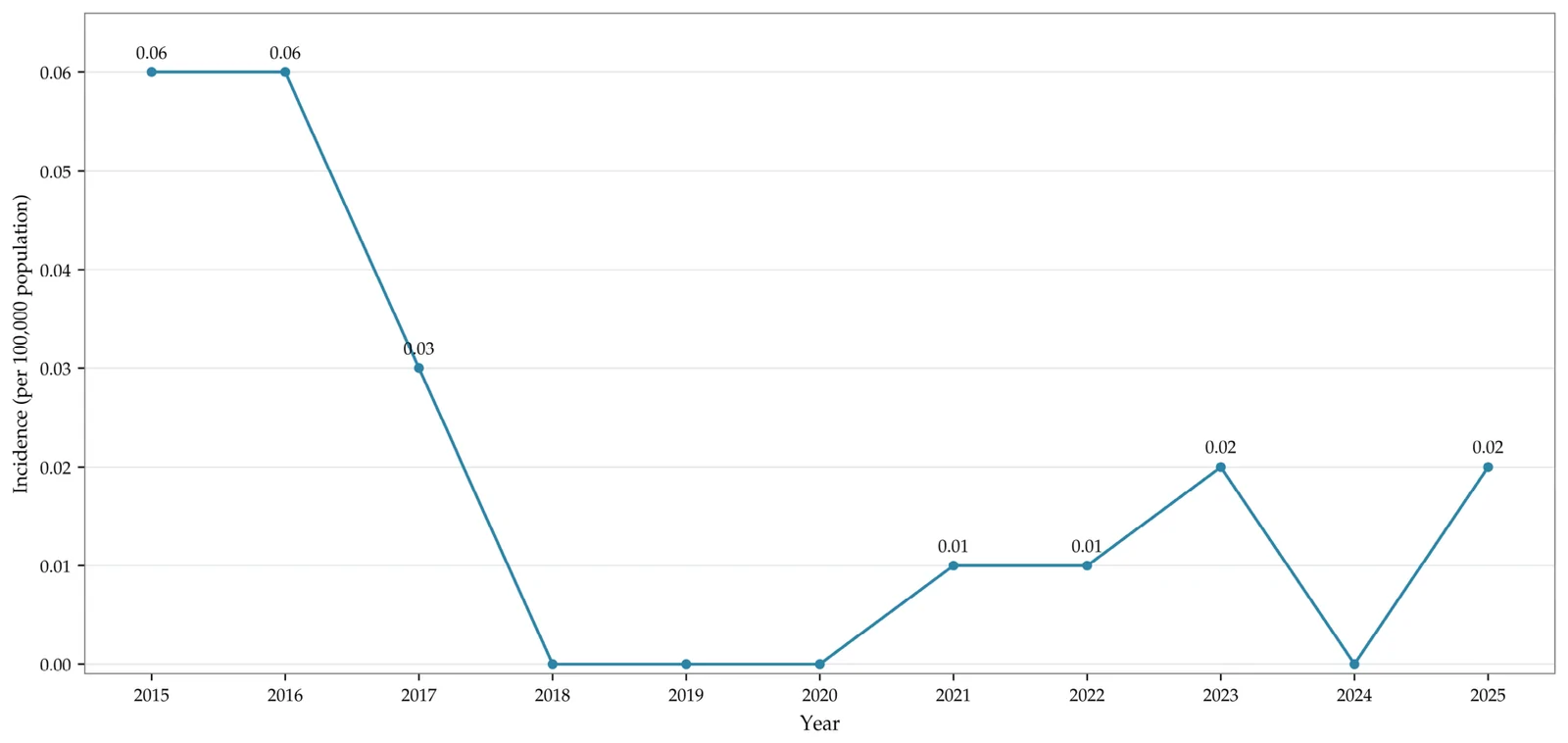
Annual incidence of reported tetanus cases per 100,000 population in Serbia, 2015–2025. Note: Data for 2024–2025 were provided by the Institute of Public Health of Serbia “Dr Milan Jovanović Batut” (official data).

The annual incidence ranged from 0 to 0.06 per 100,000 population, with the highest incidence recorded in 2015 and 2016 (0.06 per 100,000), while fewer cases were reported in subsequent years (Figure 1).

**Figure 2:**
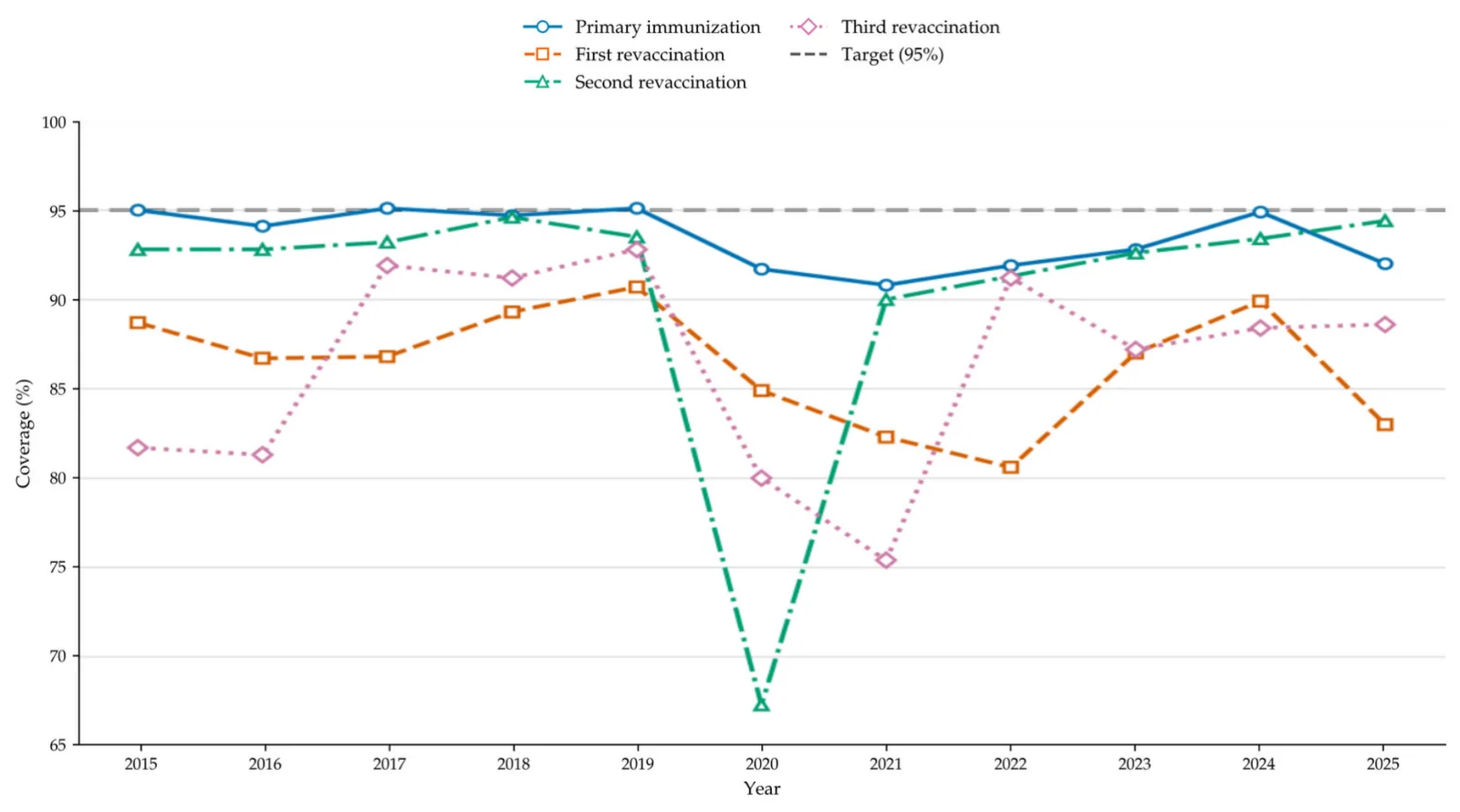
Coverage with primary immunization and revaccination using tetanus toxoid-containing vaccines in Serbia, 2015–2025. Note: Data for 2024–2025 were provided by the Institute of Public Health of Serbia “Dr Milan Jovanović Batut” (official data).

During the study period, coverage with primary immunization and revaccination using tetanus toxoid-containing vaccines varied across years and vaccination stages. Primary immunization coverage remained generally higher than that for revaccination, while the target of 95% was reached only in selected years (Figure 2). Coverage with all three revaccinations remained below the 95% target throughout the study period (Figure 2).

### 3.2. Clinical Characteristics of Pediatric Tetanus Cases

During the study period, two pediatric patients with generalized tetanus were treated at our tertiary referral center. Neither child had received a tetanus-containing vaccine, but they differed in the underlying reasons for absent immunization and in their initial clinical presentation.

#### 3.2.1. Case 1

An 18-month-old boy weighing 9.4 kg presented with fever, ear pain, and purulent discharge from the left ear. On the second day of illness, he was evaluated by a pediatrician. Laboratory inflammatory markers were within normal limits. Based on the clinical presentation, acute otitis media was suspected, and oral amoxicillin (40 mg/kg per dose, twice daily) was initiated. Otoscopy revealed an opaque left tympanic membrane without hyperemia and with an absent light reflex; however, the presence or absence of tympanic membrane perforation was not documented.

Later that day, while the child was still febrile, he experienced the first episode characterized by generalized stiffening and upward deviation of the eyes. On the third day of illness, he became afebrile but experienced three further episodes and was admitted to our hospital. According to the mother, she was unable to establish contact with the child during these episodes. At admission, the episodes were considered possible seizures in the context of the preceding febrile illness.

The patient was referred to our clinic, where he exhibited jaw stiffness, opisthotonus, and neck muscle rigidity. He was the 14th child in the family, born after an uneventful pregnancy. The child lived in adverse socioeconomic conditions in a large family and had no established contact with the healthcare system. His vaccination history was limited to BCG and hepatitis B vaccination at birth. A minor abrasion on the left index finger of unknown duration was documented in a subsequent clinical note.

Given the preceding febrile illness, the recurrent episodes, and the findings of neck muscle rigidity, meningoencephalitis was considered an important differential diagnosis. The boy was immediately transferred to the pediatric intensive care unit (PICU) for further evaluation and management. Initial laboratory evaluations, including blood tests and cerebrospinal fluid (CSF) analysis, were within normal limits. Empirical antimicrobial therapy with ceftriaxone (50 mg/kg twice daily), acyclovir (20 mg/kg every 8 h), and erythromycin (25 mg/kg every 6 h) was initiated, together with anti-edema treatment with mannitol (0.5 mg/kg every 6 h) and dexamethasone (0.1 mg/kg every 6 h), followed by methylprednisolone (20 mg/kg per day) for five consecutive days. Anticonvulsant therapy with phenobarbital (4 mg/kg per day) was also administered. Computed tomography (CT) and magnetic resonance imaging (MRI) of the brain revealed no abnormalities. Additional blood and cerebrospinal fluid studies for common causes of encephalitis were also negative. Repeated electroencephalograms (EEGs) were normal.

During the subsequent course of hospitalization, the clinical picture evolved with persistent muscle hypertonia and recurrent episodes of agitation accompanied by trismus and opisthotonus. These episodes were associated with marked discomfort and tachycardia. Between episodes, he was drowsy but easily arousable and appeared alert, with painful facial expression. No coma or persistent disturbance of consciousness was documented. He did not develop risus sardonicus, hypersalivation, apnea, respiratory muscle weakness, or persistent autonomic instability. In view of the progressive development of characteristic clinical features, and the child’s incomplete vaccination history, generalized tetanus was suspected on clinical grounds.

On the fifth day of hospitalization, the patient received an initial dose of human tetanus immunoglobulin (500 IU), followed by a full therapeutic dose of 3000 IU the next day. Episodes of agitation and opisthotonus were managed with benzodiazepines (Diazepam up 2.5 mg every 6 h), chloral hydrate up to 53 mg/kg per day, haloperidol 0.2 mg/kg per day, and baclofen 2.5 mg every 8 h. Antimicrobial therapy with metronidazole (7.5 mg/kg every 8 h) was administered, and antiviral therapy was discontinued after ten days.

Following these interventions, the patient showed gradual and significant clinical improvement. Episodes of trismus and opisthotonus progressively decreased in frequency and had ceased by day 18 of treatment. The child was alert, responded appropriately to stimuli, sat independently, stood with support, and tolerated oral intake. Sedative therapy was gradually tapered, and respiratory function remained stable throughout the hospitalization.

After 25 days in the PICU, the patient was transferred to the pediatric ward and continued a structured rehabilitation program. Immunization protocols were initiated. After a total of 35 days of treatment, he was discharged home in stable condition. At follow-up examination, no neurological or other physical abnormalities were detected.

#### 3.2.2. Case 2

A previously healthy 6-year-old boy, weighing 18.4 kg, presented to the emergency department with odynophagia, oral ulcerations, generalized weakness, and muscle spasms. His symptoms had begun three days earlier with involuntary biting of the tongue and buccal mucosa. He was examined by a pediatrician, who prescribed symptomatic therapy. His parents reported progressive weakness and jaw spasms that limited mouth opening during a dental examination. Over the following days, spasms extended to the limbs, causing difficulty walking. Further history revealed that approximately seven days prior to first symptoms, the child had fallen and sustained a superficial wound to the left knee that had neither been cleaned nor debrided.

Regarding his immunization status, the parents initially reported that he had been fully vaccinated according to the Serbian national immunization schedule. However, upon further clarification, it was established that he had received only the BCG and hepatitis B vaccines at birth and had not received any subsequent vaccinations due to parental refusal of further immunization.

On admission, he was alert and appeared mildly distressed, with slight trismus limiting mouth opening and mild abdominal muscle rigidity. Attempted forced opening of the mouth revealed hematomas on the tongue and buccal mucosa. His neck was stiff with reduced mobility. Physical examination of the previously injured knee revealed a 2 × 2 cm laceration covered with a dry crust.

Within the subsequent three hours, he developed generalized spasms of the extremities and abdominal musculature, accompanied by severe trismus. The combination of trismus, neck stiffness, and abdominal muscle rigidity, together with the history of a recent wound and the subsequently clarified absence of tetanus immunization, raised a strong clinical suspicion of tetanus. The rapid progression to generalized spasms further supported this diagnosis. Head CT and video EEG were performed to rule out alternative diagnoses. Following laboratory and microbiological assessment, he received 3000 IU of intramuscular human tetanus immunoglobulin and tetanus vaccine and started on intravenous metronidazole (7.5 mg/kg every 8 h) and ceftriaxone (50 mg/kg twice daily).

Due to progressive muscle spasms involving the respiratory muscles and impending respiratory failure, the patient underwent fiberoptic endotracheal intubation, which was challenging because of severe trismus, and mechanical ventilation was initiated. Rocuronium bromide (up to 0.5 mg/kg per hour) was administered for neuromuscular blockade. Supportive therapy included continuous midazolam infusion with maximal infusion rate 0.1 mg/kg per hour, intermittent parenteral diazepam (5 mg every 6 h), and oral baclofen (5 mg every 8 h) for more muscle relaxation and sedation.

During the first week of PICU treatment, he developed severe autonomic dysfunction, with tachycardia up to 200 beats per minute and arterial blood pressure up to 180/100 mmHg. Continuous labetalol infusion (with maximal infusion rate of 0.5 mg/kg per hour) was initiated to stabilize hemodynamics, while continuous magnesium sulfate infusion (9 mg/kg per hour) was administered to help control autonomic instability and to provide more muscle relaxation. Nutritional support was kept via nasogastric tube feeding.

Gradual clinical improvement was seen following these therapeutic measures. Autonomic instability resolved within five days, allowing discontinuation of the labetalol infusion. Muscle spasms became shorter and less frequent, and sedative and neuromuscular blocking agents were progressively reduced and stopped: first rocuronium and then midazolam, diazepam, and finally baclofen.

He was extubated after 12 days of mechanical ventilation, and nasogastric feeding was discontinued after 14 days as oral intake was reintroduced. After 18 days in the PICU, he was transferred to the pediatric ward for continued rehabilitation. The patient was discharged home after 25 days of hospitalization, in stable condition, with mild hypertonia, which was resolved with rehabilitation.

The clinical characteristics, vaccination history, management, and outcomes of both patients are presented in Table 2.

## 4. Discussion

### 4.1. Changing Epidemiology of Tetanus and the Impact of Immunization

Routine childhood vaccination against tetanus has dramatically reduced the global burden of the disease, resulting in a substantial decline in tetanus incidence and mortality [1,11]; Nevertheless, sporadic cases continue to occur even in high-income countries.

In the United States, 402 tetanus cases and 37 associated deaths were reported between 2009 and 2023, with cases occurring predominantly among older adults [11]. Similarly, surveillance data from the European Union/European Economic Area indicate that most reported tetanus cases occurred among individuals aged ≥65 years. In 2024, 27 countries submitted surveillance data: 16 reported no cases, while 11 reported a total of 73 cases, including six among children aged 5–14 years [22].

The data from Serbia presented in our study showed a similar pattern, with tetanus cases occurring predominantly among older adults (Table 1). This age distribution may reflect incomplete protection among previous generations and insufficient uptake of recommended booster doses. Protection against tetanus depends on completion of the primary vaccination series and maintenance of immunity through recommended booster doses; incomplete or delayed booster vaccination may result in insufficient or waning immunity [1,11,23].

Neonatal tetanus was not reported during the study period. According to national surveillance data, the last reported case of neonatal tetanus in Serbia occurred in 2009 [21].

Although childhood vaccination coverage remains high across Europe, recent declines in coverage raise concerns regarding increasing numbers of individuals with incomplete protection [22]. In Serbia, primary vaccination coverage with tetanus-containing vaccines varied during the analyzed period, while booster vaccination coverage remained below the 95% target (Figure 2), indicating persistent gaps in individual protection. Unlike vaccine-preventable diseases transmitted from person to person, tetanus does not spread between individuals; therefore, population-level immunity does not protect unvaccinated persons, and individual vaccination remains the primary means of prevention [1,2].

The occurrence of the disease in two children who had not received a tetanus-containing vaccine further illustrates that, although rare, pediatric tetanus can still occur when vaccination is not initiated or the recommended schedule is not completed. Unvaccinated children remain susceptible to disease even after apparently minor wounds, emphasizing the significance of maintaining high vaccination coverage and ensuring timely catch-up immunization [1,2,11].

### 4.2. Vaccination Status and Diagnostic Implications

The two cases illustrate distinct pathways to lack of tetanus immunization: limited access to healthcare in the setting of socioeconomic vulnerability and lack of established contact with healthcare services in Case 1, and explicit parental refusal of further vaccination in Case 2. These contrasting circumstances highlight the importance of addressing both barriers to healthcare access and parental refusal when seeking to improve childhood immunization coverage [12,13].

The role of vaccination history differed between the two cases. In the first patient, the absence of immunization against tetanus had been documented, but its diagnostic significance was not appreciated during the initial evaluation because the clinical presentation strongly suggested meningoencephalitis. Its relevance became apparent only after further evaluation when the evolving clinical picture proved to be more consistent with generalized tetanus. In the second patient, the absence of tetanus vaccination was disclosed only after repeated questioning.

These cases emphasize that careful assessment of vaccination status should be an integral part of the evaluation of children presenting with unexplained neuromuscular symptoms. When the immunization history is incomplete or uncertain, it should be actively verified rather than assumed, particularly in the presence of a wound or another potential portal of entry [2,10,16].

### 4.3. Atypical Clinical Presentation and Diagnostic Challenges

Both of our cases illustrate the diagnostic complexity of early tetanus, although through markedly different clinical presentations.

In the first patient, fever and otorrhea initially favored an infectious process, while the recurrent episodes of generalized stiffening raised the possibility of seizures. Meningoencephalitis was therefore considered in the differential diagnosis. However, cerebrospinal fluid analysis and neuroimaging did not provide evidence supporting central nervous system infection, and repeated electroencephalographic examinations were normal. The diagnosis of generalized tetanus was ultimately established on clinical grounds as characteristic features, particularly trismus and opisthotonus, became increasingly evident in an unimmunized child. This case illustrates that tetanus may not be readily recognized at the initial assessment and that the diagnosis may become apparent only as characteristic clinical signs evolve.

The portal of entry in the first patient also remained uncertain. Acute otitis media was suspected at the initial evaluation because of fever, ear pain, and purulent discharge from the left ear. However, the available clinical documentation was insufficient to establish the precise nature of the ear infection, as the integrity of the tympanic membrane was not recorded. Therefore, although the ear could have represented a possible portal of entry for *Clostridium tetani*, this could not be confirmed. A minor abrasion on the left index finger, documented in a subsequent clinical note, represented another possible portal of entry. The duration of the ear symptoms before presentation and the duration of the abrasion were not known with certainty.

The second child initially presented with odynophagia and repeated involuntary tongue biting resulting in oral trauma, prompting evaluation by both a pediatrician and a dentist before tetanus was suspected. Dysphagia and odynophagia have been reported as early manifestations of tetanus in both children and adults and may lead to consideration of more common oropharyngeal or dental conditions [15,24,25]. Recognizing these nonspecific early manifestations is important because delayed diagnosis may allow disease progression, potentially resulting in respiratory compromise and autonomic dysfunction.

In contrast to the first patient, the tetanus was recognized immediately upon admission in the second case. Although the reasons for this difference cannot be determined with certainty, previous clinical experience with a similar presentation may have contributed to earlier recognition. This observation underscores the importance of continued clinical education and vigilance for diseases that are rarely encountered but may have severe consequences when diagnosis and treatment are delayed.

Thus, the declining incidence of paediatric tetanus should not lead to its exclusion from the differential diagnosis when vaccination is absent or uncertain and compatible neuromuscular features are present.

### 4.4. Management

Once tetanus is suspected, prompt and individualized management is essential. Treatment requires a multidisciplinary approach, including wound care, administration of human tetanus immunoglobulin, appropriate antimicrobial therapy, control of muscle spasms, and supportive care [2,10,17,26,27]. Severe cases may require intensive care with sedation, airway protection, nutritional support, and careful management of autonomic instability [2,10,17].

The clinical course of our two patients illustrates the variability in disease severity and the need to adapt treatment to the patient’s evolving condition. One developed respiratory failure and severe autonomic dysfunction, requiring mechanical ventilation, neuromuscular blockade, and hemodynamic support. The other had no respiratory or significant autonomic involvement, but experienced prolonged muscle spasms requiring intensive sedation and extended PICU management. Despite these differences, both patients improved following tetanus-specific therapy and supportive intensive care, highlighting the importance of early recognition and timely initiation of treatment.

Both children initiated catch-up immunization during hospitalization. Because tetanus infection does not confer protective immunity, completion of the recommended vaccination schedule remains necessary after recovery and should be considered an integral part of post-tetanus care and prevention of future disease [1,2]. This may be challenging, however, as misconceptions about naturally acquired immunity and vaccine safety can affect adherence even after recovery. Previous reports have described children who did not complete vaccination following severe tetanus requiring intensive care [28]. In this context, our cases reinforce the importance of effective communication with families and appropriate follow-up to support adherence to recommended vaccination.

### 4.5. Proposed Clinical Framework for Timely Recognition of Paediatric Tetanus

**Figure 3:**
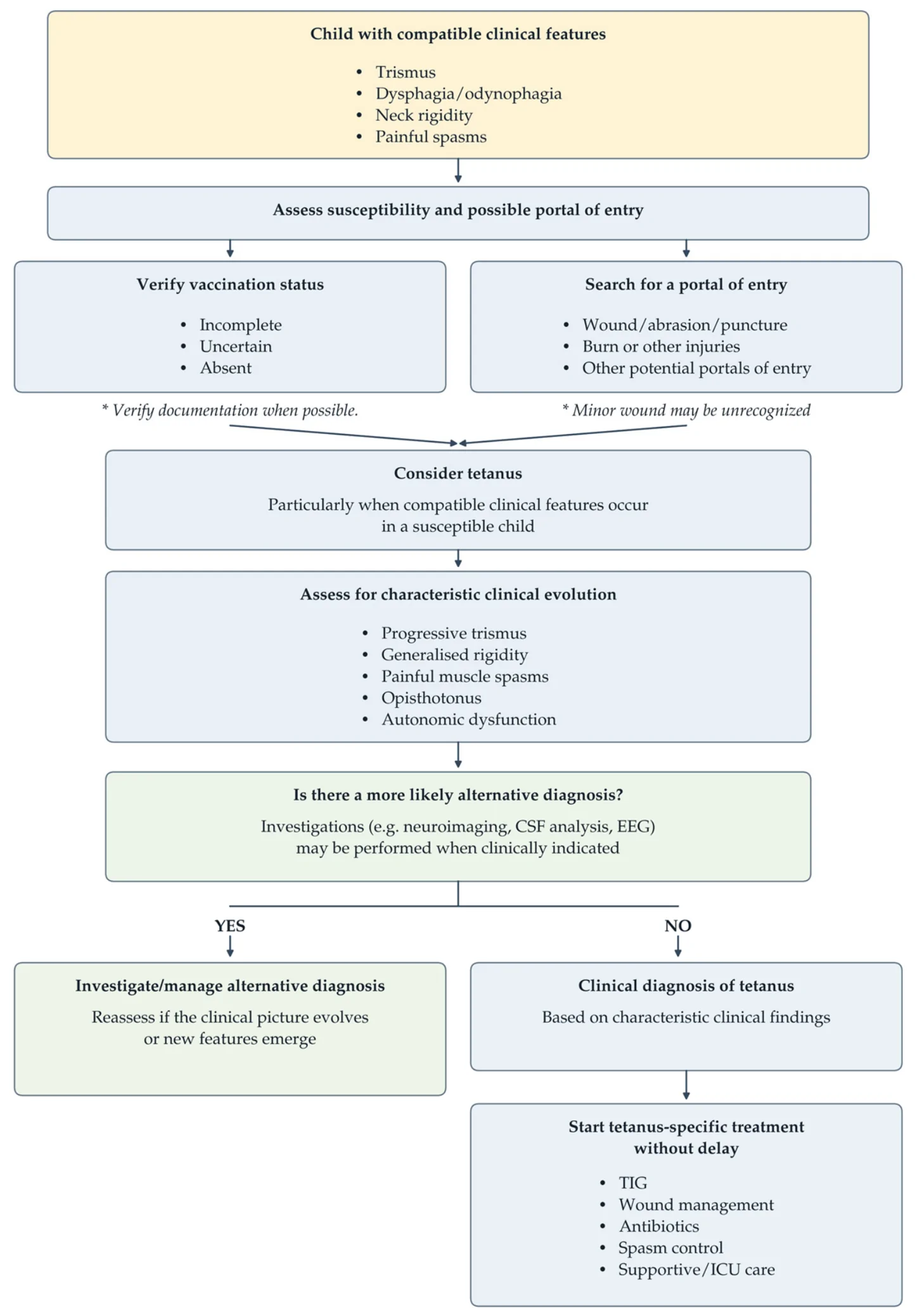
Proposed clinical framework for timely recognition of paediatric tetanus. Legend: CSF—cerebrospinal fluid; EEG—electroencephalography; ICU—Intensive Care Unit; TIG—tetanus immunoglobulin. Note: The asterisk (*) indicates an explanatory note providing additional clarification regarding vaccination history and the possibility of a minor or unrecognized portal of entry.

Based on the clinical trajectories of the two patients and our previous experience with paediatric tetanus in Serbia, we propose a practical clinical framework for its timely recognition (Figure 3). Tetanus should be considered in any child presenting with unexplained trismus, muscle rigidity, recurrent painful spasms, opisthotonus, or progressive oropharyngeal symptoms, particularly when these manifestations are unusual for the initially suspected diagnosis. Assessment should then include two key elements: verification of tetanus-containing vaccination status and active inquiry about a recent wound or other potential portal of entry. Importantly, the absence of a recognized wound should not exclude tetanus, as the portal of entry may be minor or its timing may be unknown.

When the presentation is atypical, the diagnosis should be reconsidered as characteristic features evolve. Common alternative diagnoses should be evaluated according to the clinical presentation; however, normal or non-supportive investigations should prompt reassessment rather than reassurance when clinical features suggest tetanus. In particular, repeated or stimulus-associated muscle spasms, progressive trismus, generalized rigidity, and opisthotonus should increase the level of suspicion. Because tetanus is primarily a clinical diagnosis, treatment should not be delayed while awaiting laboratory confirmation.

This approach may be particularly valuable in settings where paediatric tetanus has become rare and clinicians may have limited recent experience with its presentation.

### 4.6. Public Health Perspectives

National immunization programs have markedly reduced the burden of tetanus. Our epidemiological overview, together with the two pediatric cases, shows that susceptible individuals remain at risk in the presence of immunization gaps. Most reported tetanus cases occurred among older adults whose childhood immunization period preceded that of the cohorts covered by the vaccination data analyzed here. Nevertheless, childhood vaccination coverage presented in our study is relevant to understanding the two pediatric cases, as both children belonged to birth cohorts covered by the mandatory routine immunization programs during the study period. The observed variation in vaccination coverage over the study period points to persistent gaps in routine immunization and a continued risk of tetanus among children who are unvaccinated or incompletely vaccinated. These findings should not be interpreted as an explanation for the overall age distribution of tetanus cases, but rather as an indication that risk of pediatric tetanus persists among unvaccinated or incompletely vaccinated children. The different circumstances leading to non-immunization in our patients indicate that social vulnerability and parental vaccine refusal are among the reasons for missed vaccination. Preventing tetanus in children therefore requires both maintaining high vaccination coverage and identifying vaccination gaps early enough to initiate or complete catch-up vaccination.

## 5. Conclusions

Although tetanus has become uncommon in countries with successful immunization programs, these cases demonstrate that pediatric tetanus remains a consequence of missed vaccination opportunities. Combined with our epidemiological findings, they reinforce the need for sustained immunization efforts and remind clinicians that rare vaccine-preventable diseases should remain in the differential diagnosis in unvaccinated children with compatible clinical features.

Our experience also illustrates that pediatric tetanus is not a uniform clinical entity. Initial manifestations, disease severity, and treatment requirements differed substantially between the two children, influencing both the diagnostic process and the intensity of supportive care. Nevertheless, favorable outcomes were achieved in both cases, reinforcing the importance of early recognition, prompt tetanus-specific therapy, and access to experienced multidisciplinary intensive care.

## Figures and Tables

**Table 1:** Distribution and characteristics of reported tetanus cases in Serbia, 2015–2025 (*n* = 14).

Characteristic	*n* (%)
Year of diagnosis	
2015	4 (28.6)
2016	4 (28.6)
2017	2 (14.3)
2021	1 (7.1)
2022	1 (7.1)
2023	1 (7.1)
2025	1 (7.1)
Sex	
Female	10 (71.4)
Male	4 (28.6)
Age group	
1–9 years	2 (14.3)
20–29 years	1 (7.1)
≥60 years	11 (78.6)
Region	
Central Serbia	12 (85.7)
Vojvodina	2 (14.3)
Tetanus vaccination status	
Unvaccinated	2 (14.3)
Unknown	12 (85.7)
**Outcome**	
Survived	8 (57.1)
Died	6 (42.9)

Note: No tetanus cases were reported in 2018–2020 or 2024. Data for 2024–2025 were provided by the Institute of Public Health of Serbia “Dr Milan Jovanović Batut” (official data).

**Table 2:** Clinical characteristics, vaccination history, management, and outcomes of two pediatric patients with generalized tetanus.

Parameter	Case 1	Case 2
Clinical characteristics and vaccination history		
Age	18 months	6 years
Vaccination status	No tetanus-containing vaccines	No tetanus-containing vaccines
Reason for lack of tetanus immunization	Socioeconomic vulnerability	Parental refusal
Initial vaccination history	Known, but initially not considered relevant	Not initially disclosed; clarified on further questioning
Initial symptoms	Fever, ear pain, otorrhea; episodic rigidity and ocular deviation	Odynophagia, oral ulcerations, involuntary bites, progressive spasms
Diagnostic challenges	Initially suggestive of ME	Nonspecific early symptoms
Characteristic tetanus signs	Trismus, opisthotonus, hypertonia, episodic agitation	Severe trismus, generalized spasms, autonomic dysfunction
Autonomic dysfunction	No significant autonomic dysfunction	Severe tachycardia and hypertension
Management and outcome		
Human tetanus immunoglobulin (HTIG)	500 IU IM + 3000 IU IM	3000 IU IM
Antimicrobial therapy	Metronidazole (after initial empirical therapy for ME)	Metronidazole + ceftriaxone
Sedation and spasm control	Benzodiazepines, chloral hydrate, haloperidol, baclofen	Benzodiazepines, baclofen
Neuromuscular blockade	None	Rocuronium
Mechanical ventilation	No	Yes
Hemodynamic/autonomic support	No	Yes, labetalol, magnesium sulphate
Catch-up immunization	Yes, initiated during PICU stay	Yes, initiated during PICU stay
Outcome	Full recovery	Full recovery

Legend: ME, meningoencephalitis; PICU, Pediatric Intensive Care Unit.

## Data Availability

Data for 2015–2023 are publicly available in the official reports cited in the reference list. Official epidemiological data for 2024 and 2025 were provided by the Institute of Public Health of Serbia “Dr Milan Jovanović Batut” and are available from the corresponding author upon reasonable request, with permission from the data provider. The clinical data supporting the findings of this study were obtained from the medical records of the Mother and Child Health Care Institute of Serbia “Dr Vukan Čupić” and are not publicly available due to patient privacy and ethical restrictions.

## Abbreviations

The following abbreviations are used in this manuscript:

ICD	International Classification of Diseases
BCG	Bacillus Calmette–Guérin
PICU	Pediatric Intensive Care Unit
CSF	Cerebrospinal fluid
CT	Computerized tomography
MR	Magnetic resonance
EEGs	Electroencephalograms
HTIG	Human tetanus immunoglobulin
ME	Meningoencephalitis
